# Stellate Ganglion Blockade repairs Intestinal Mucosal Barrier through suppression of Endoplasmic Reticulum Stress following Hemorrhagic Shock

**DOI:** 10.7150/ijms.47662

**Published:** 2020-07-30

**Authors:** Meng Yin, Zhong-Hua Li, Chen Wang, Ying Li, Hong Zhang, Hui-Bo Du, Zhen-Ao Zhao, Chun-Yu Niu, Zi-Gang Zhao

**Affiliations:** 1 Institute of Microcirculation, Hebei North University, Hebei Zhangjiakou, PR China.; 2 Hebei Medical University, Shijiazhuang, PR China.

**Keywords:** Stellate ganglion blockade, Hemorrhagic shock, Endoplasmic reticulum stress, Intestinal mucosal barrier

## Abstract

**Background:** Hemorrhagic shock-induced ischemia and hypoxia elicit endoplasmic reticulum stress (ERS) that leads to cell apoptosis, tissue structural damage and organ dysfunction and failure. Stellate ganglion blockade (SGB) has been demonstrated to improve intestinal barrier dysfunction induced by hemorrhagic shock. The present study sought to investigate whether the beneficial effect of SGB on the intestinal mucosal barrier function is via suppression of ERS.

**Materials and methods:** A conscious rat model of hemorrhagic shock (40 ±2 mmHg for 1 hour, followed by resuscitation) was established. The parameters reflecting intestinal morphology and intestinal mucosal barrier function including wet-dry ratio (W/D), intestinal permeability, D-lactic acid (D-LA) and intestinal fatty acid binding protein (I-FABP) in plasma, and expressions of ATF6α, PERK, and IRE1α in intestinal tissues were then observed. Furthermore, the effects of either SGB or ERS inhibitor, 4-phenylbutyric acid (4-PBA), on these parameters in rats with hemorrhagic shock were assessed. The effect of ERS agonist tunicamycin (TM) on the rats subjected with both SGB and hemorrhagic shock was also determined.

**Results:** Either SGB or administration of ERS inhibitor, 4-PBA, alleviated hemorrhagic shock-induced adverse effects such as intestinal mucosal barrier dysfunction and excessive autophagy, which were characterized by damaged intestinal tissue, enhanced intestinal permeability and D-LA and I-FABP levels in plasma, and increased expressions of ATF6α, PERK, IRE1α in intestinal tissue. In contrast, administration of ERS agonist, TM, suppressed the beneficial effects of SGB on intestinal tissue and function during hemorrhagic shock.

**Conclusion:** The SGB repairs intestinal mucosal barrier through suppression of ERS following hemorrhagic shock.

## Introduction

Hemorrhagic shock is a leading cause of sudden death for severe multiple trauma and hemorrhage patients 1. Severe hemorrhagic shock leads to multiple organ dysfunctions through ischemia-reperfusion injury and systemic inflammatory responses 2.

The normal function of intestinal barrier is essential for the stability of the internal environment and intestinal mucosal barrier is the most important structure of intestinal barrier. However, the intestinal tract contains a high content of bacteria and is vulnerable to ischemic damage that causes intestinal barrier dysfunction and bacteremia 3. The injury of intestinal mucosal barrier after hemorrhagic shock would elicit severe intestinal injury and dysfunction, inducing translocation of intestinal pathogens and endotoxin. Therefore, intestinal mucosal barrier injury is the first gateway leading to intestinal barrier injury 4, and has been considered as a critical mechanism responsible for uncontrolled systemic inflammatory response.

Stellate ganglion block (SGB) is widely used in clinical analgesia. Previous study 5 demonstrated that SGB can reduce intestinal injury and improve the survival condition of animals after hemorrhagic shock. Also, sympathetic transection simulated by SGB reduces organ injury in septic rats 6. However, the mechanism of SGB attenuation of intestinal injury after hemorrhagic shock is still unclear. Recent studies have shown that excessive endoplasmic reticulum stress (ERS) participates in the process of intestinal mucosal epithelial cell injury after hemorrhagic shock, suggesting that ERS may be an important contributor to intestinal mucosal barrier injury. However, whether the inhibitory effect of SGB on intestinal injury induced by hemorrhagic shock is associated with inhibition of the ERS remains unknown. Therefore, we hypothesized that the beneficial effect of SGB on intestinal mucosal barrier injury is via inhibition of the ERS. To test this hypothesis, the effects of administration of either the ERS inhibitor, 4-phenylbutyric acid (4-PBA), or agonist, tunicamycin (TM) in rats with or without SGB during hemorrhagic shock were determined. The molecular mechanisms related to the effects of ERS inhibitor, agonist and SGB were also explored in collected intestinal tissues.

## Materials and Methods

### Experimental animals

Thirty-six male Wistar rats were purchased from Sibef Biotechnology Co., Ltd (Beijing, China). The animals were housed in SPF environment. All surgery procedures were approved by the Animal ethics committee of Hebei North University. All animal were then subjected to hemorrhagic shock or sham operation at 4-5 months of ages, weighing 300±20 g.

### Experimental procedures

The rats were divided into the Sham, Shock, Sham+SGB, Shock+SGB, Shock+4-phenylbutyric acid (4-PBA) (Shock plus 4-PBA treatment), and Shock+SGB+tunicamycin (TM) (Shock+SGB plus TM administration) groups, with n=6 for each group. According to the methods in our lab 5, right SGB was performed before hemorrhagic shock. In the SGB group, 0.25% ropivacaine hydrochloride (AstraZeneca AB, Sweden 2018-05 2021-04 LBKT) in 0.2 mL saline was injected into the body surface landmarks of the right stellate ganglion after isoflurane inhalation anesthesia, and the rats were naturally awakened to observe whether there was Horner syndrome. Rats without SGB were administered with an equal volume of physiological saline. Then, the hemorrhagic shock was induced in the Shock, Shock+SGB, Shock+4-PBA and Shock+SGB+TM groups. In the Sham and Sham+SGB groups, same operation was performed without hemorrhage and resuscitation. In addition, subcutaneous injections of 4-PBA (5 μg/kg, Merck KGaA, Darmstadt, Germany) or TM (2 mg/kg, ApexBio, Texas, USA) were performed at the same time of fluid resuscitation in the Shock+4-PBA or Shock+SGB+TM groups, respectively.

### Conscious rat model of hemorrhagic shock

Rats received inhalation anesthesia of isoflurane (Shenzhen Ruiwald Life Technology Co., Ltd., Shenzhen, China) using the inhalation anesthesia machine for small animals (MATRX VMR, Midmark Corporation, Dayton, Ohio), and underwent femoral surgery for hemorrhagic shock. Briefly, bilateral femoral artery and vein were separated and intubated into the femoral artery and vein respectively. Then, the intubation was drawn from the middle of the two shoulder blades of the neck through the subcutaneous tunnel of the back, and the tube was sealed with heparin after fixation. The right femoral artery was cannulated to monitor the mean arterial pressure (MAP) using the PowerLab biological signal acquisition system (ADInstruments, Bella Vista NSW, Australia), and the left femoral artery was intubated for blood withdrawal. After all the operations, the inhalation anesthesia was relieved for natural awake within 1 to 3 minutes. Then, after a 20-min stabilized phase, blood was withdrawn and MAP reached 40±2 mmHg within 10 minutes, which was maintained at this level for 60 minutes through pumping or infusing the withdrawal blood as required using the automatic withdrawal-infusion machine (NE-1000, New Era Pump Systems Inc., Farmingdale, NY). During the hypotension, the shed blood stored in a 20 ml-syringe connected automatic withdrawal-infusion machine, at room temperature. After 1 hour of hypotension, intravenous infusion of the shed whole blood plus an equal volume of Ringer's solution was carried out within 30 minutes for resuscitation.

### D-lactic acid (D-LA) and intestinal fatty acid binding protein (I-FABP) concentrations

After resuscitation for 3 hours in hemorrhagic shocked rats or corresponding time points in sham operation rats, the rats received inhalation anesthesia of isoflurane and general anesthesia with 1% pentobarbital sodium (50 mg/kg, Merck, Germany). Then, inferior vena cava blood was harvested and centrifuged at 3000 rpm for 10 minutes. The D-LA and I-FABP levels in the plasma were detected using the rat-specific enzyme-linked immunosorbent assay kits (Wuhan Pure Biology Co., Ltd. HuBei, China) as the manufacturer's protocols.

### Collection of intestinal tissues

The fresh intestinal segments located on 15 cm upwards from the ileocecal junction were obtained at the end of blood samples collection, and rinsed with the phosphate buffer saline (PBS) solution to remove the bowel contents. Then, starting in the ileocecal junction, the intestinal segment from each rat was cut into four sections, 6 cm, 2 cm, 2 cm, and 5 cm, respectively. These intestinal segments were used for detections of intestinal permeability, wet-dry ratio (W/D), morphology, protein biomarkers of ERS, respectively.

### Intestinal permeability

The intestinal sac was turned over and ligated with 4 # thread at the end, and the other end was ligated after the injection of 0.3 ml's KH solution. Then, the overturned segment of the intestine was incubate in KH solution containing the fluorescein isothiocyanate-dextran 4 (FD4, Sigma) with a concentration of 25 mg/ml for 30 minutes, maintain the temperature at 37 °C, and continuously fill with a mixture of 95% oxygen and 5% carbon dioxide. The intestinal solution was centrifuged at 1000 g for 10 minutes at 4 °C, and the supernatant (100 μL) was added to the 900 μL of PBS. The fluorescence intensity of FD4 in mixed liquid was measured with microplate reader (SpectraMax M3, Molecular Devices, San Jose, CA). The concentration of FD4 in intestinal sac was calculated according to this standard curve (*y*=0.0095*x*+0.4011, *R²*=0.9939). Then, the permeability of intestinal sac to FD4 was calculated with the formula as follows, which was used to evaluate the intestinal permeability.


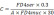


*C* represents the transmittance of intestinal tissue per unit area to FD4 (nL/(minute·cm^2^). A represents the surface area of the intestinal sac. FD4ser represents the concentration of FD4 in the intestinal sac after incubation (μg/mL). FD4muc represents the concentration of FD4 outside the intestinal sac before incubation (μg/mL). And 30 represent incubation time (minute).

### Intestinal wet-dry ratio

The intestinal tissue was placed on the weighed tin foil. Firstly, the intestinal tissue were weighed before put them into an oven at 60 °C to dry. Secondly, they were taken out after 72 hours, the dry weight was obtained. W/D = (wet weight - paper weight)/(dry weight - paper weight).

### Intestinal morphology

The fresh intestinal samples were washed with cold saline and preserved in 4% paraformaldehyde for 24 hours. Subsequently, they were dehydrated through gradient dehydration and paraffin embedding. Sections (4 μm) were cut with a microtome RM2235 (Leica Microsystems GmbH, Wetzlar, Germany) and stained with hematoxylin/eosin for histopathological evaluation, three sections from each animal. Images were acquired using an Olympus BH-2 microscope (Japan). As previous report 7, the intestinal structure was observed for the measurement of intestinal villus height and thickness of sub mucosa and muscular layer. Degrees of structural injury in intestinal tissues were analyzed.

### Expressions of ATF6α, PERK, IRE1α

The intestinal tissues (20 mg) was homogenized with 200 μL RIPA lysis containing proteinase inhibitors, and then centrifuged at 12000 rpm for 5 minutes for the collection of supernatant containing protein for further analysis. The concentrations of protein samples were determined using BCA kit (Beijing Puli Gene Technology Co., Ltd. Beijing, China). Protein samples were loaded on a 10% SDS-PAGE gel and transferred to PVDF membrane. After blocked in 5% nonfat milk for 1 hour, the membranes were incubated with the primary antibodies anti-ATF6α (ab203119, 1:1000, Abcam Inc., Cambridge, MA), anti- PERK (70R-17036, 1:1000, Fitzgerald Inc, MA) and anti-IRE1α (ab37117, 1:1000, Abcam Inc., Cambridge, MA) at 4 °C overnight. After washed three times with TBS-Tween (TBS-T), the membranes were incubated with appropriate HRP-conjugated secondary antibodies (dilution 1: 5000) at room temperature for 1 hour. Blots were developed with chemiluminescence detection reagents (Applygen Technologies Inc., Beijing, China) and imaged using ImageQuant™ LAS 4000 (General Electric Company, Boston, USA). The relative band density was qualified using Quantity One v4.6.2 software.

### Statistical analysis

All statistical analyses were performed using SPSS software. All data were expressed as mean ± SEM. One-way analysis of variance (ANOVA) was used to determine the significance of the differences between groups, followed by LSD test as a post hoc test for multiple comparisons. *P*<0.05 was considered as statistically significance.

## Results

### Changes of MAP in rats after hemorrhagic shock

**Figure 1:**
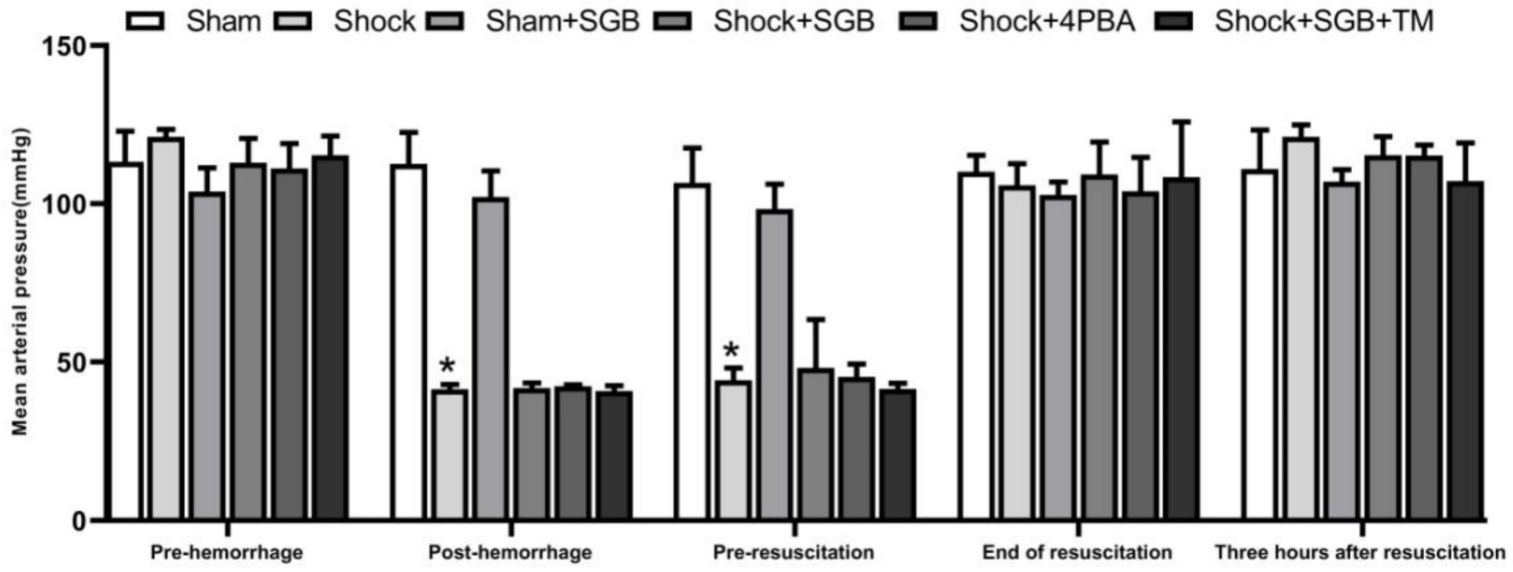
Changes of MAP in rats after hemorrhagic shock. Data are presented as mean ± SEM, with n = 4-6 for each group. *P < 0.05 vs. the Sham group at the same time.

**Figure 1** showed that there were no significant differences in MAP between the Sham and Sham+SGB groups. Also, there were no statistic differences in MAP among the Shock, Shock+SGB, Shock+4-PBA, and Shock+SGB+TM groups at different time periods including pre-hemorrhage, post-hemorrhage, pre-resuscitation, end of resuscitation, and three hours after resuscitation. However, at the time periods of post-hemorrhage and pre-resuscitation, the MAP in the Shock, Shock+SGB, Shock+4-PBA, and Shock+SGB+TM groups were significantly decreased when compared to the Sham group. At the end of resuscitation and three hours after resuscitation, three were no statistic differences in MAP among these six groups.

### Effects of SGB and ERS related tool drugs on intestinal injury in rats after hemorrhagic shock

**Figure 2:**
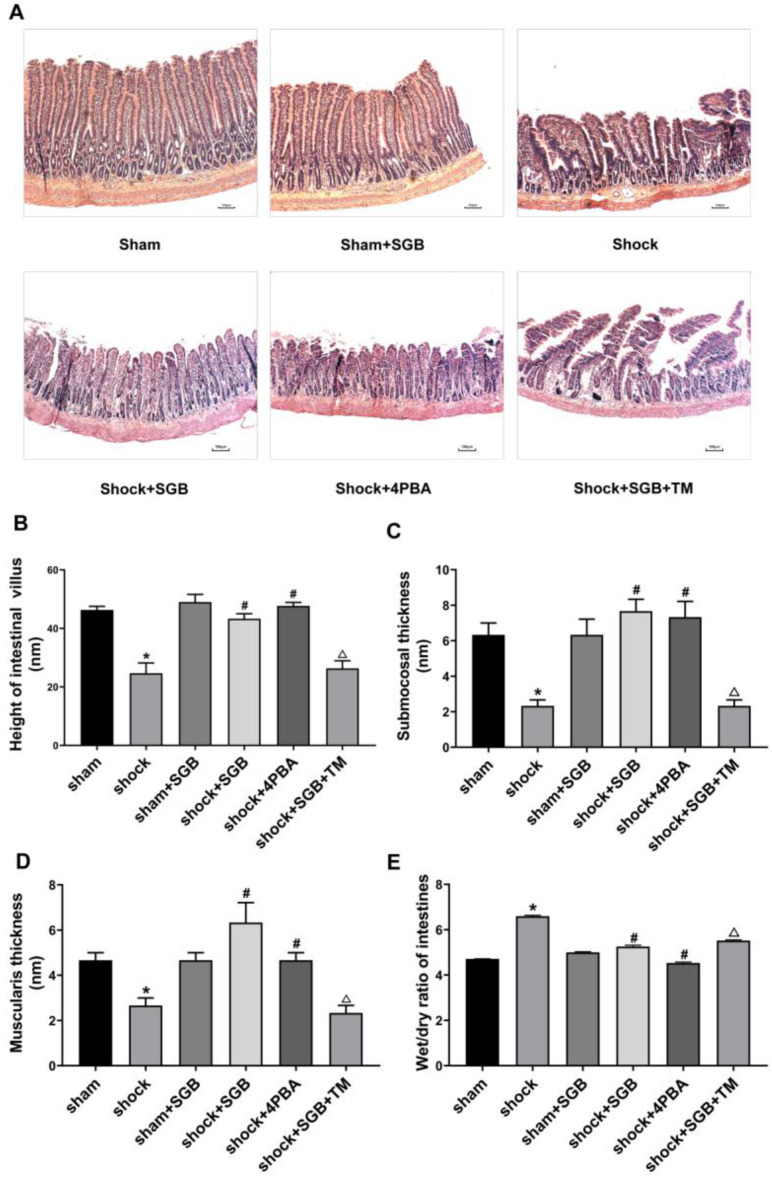
** Role of ERS in SGB alleviating hemorrhagic shock induced-intestinal injury in rats.** (**A**) Characteristic images of intestinal histopathology (HE staining, Bar = 100 µm); (**B**) Height of intestinal villus; (**C**) Submucosal thickness; (**D**) Muscularis thickness; (**E**) Wet/dry ratio (W/D) of intestines. Data are presented as mean ± SEM, with n = 3 for each group. The analysis of ANOVA showed that there was statistical difference among these six groups, followed by LSD test as follows: *P < 0.05 vs. the Sham group, ^#^*P* < 0.05 vs. the Shock group, ^Δ^*P* < 0.05 vs. the Shock+SGB group.

Shock and Shock+SGB groups showed short, irregular and fractured intestinal villus and obvious morphologic damage compared with the Sham and Sham+SGB groups. Furthermore, the injury of intestinal structure in Shock+SGB+TM group was more serious than that in the Shock+SGB group (**Figure 2A**).

In the Shock group, the height of intestinal villi, the thickness of submucosa and the thickness of muscle layer were significantly lower than Sham group (*P*<0.05). SGB and 4-PBA treatment significantly enhanced the height of intestinal villi, the thickness of submucosa and the thickness of muscle layer (*P*<0.05). At the same time, in the Shock+SGB+TM group, these indices were significantly reduced compared the Shock+SGB group (*P*<0.05) (**Figure 2B-D**).

In **Figure 2E**, the W/D ratio of intestinal tissue was significantly increased in the shock group than the Sham group (*P*<0.05). SGB and 4-PBA treatment reduced the intestinal W/D in rats after hemorrhagic shock (*P*<0.05). However, TM abolished the role of SGB in reducing the intestinal W/D in rats after hemorrhagic shock (*P*<0.05).

### Effects of SGB and ERS related tool drugs on intestinal barrier in rats undergo hemorrhagic shock

**Figure 3:**
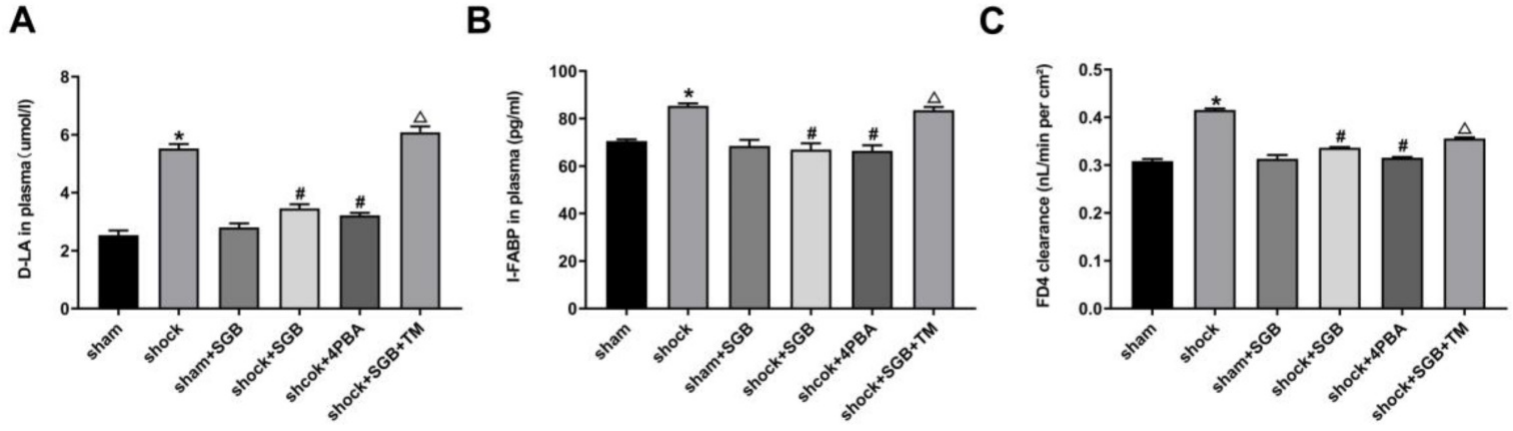
** Role of ERS in SGB alleviating hemorrhagic shock induced-intestinal barrier dysfunction in rats.** (**A-B**) Levels of D-lactic acid (D-LA) and intestinal fatty acid binding protein (I-FABP) in plasma; (**C**) Intestinal clearance of fluorescein isothiocyanate-dextran (FD4) in rats. Data are presented as mean ± SEM, with n = 6 for each group. The analysis of ANOVA showed that there was statistical difference among these six groups, followed by LSD test as follows: * *P* < 0.05 vs. the Sham group, ^#^*P* < 0.05 vs. the Shock group, ^Δ^*P* < 0.05 vs. the Shock+SGB group.

As presented in **Figure 3A-B**, the concentrations of D-LA and I-FABP in plasma obtained from the Shock group were significantly increased compared to the Sham group, (*P*<0.05), and these indices were attenuated by SGB or 4-PBA treatments. Compared with Shock+SGB group, the concentrations of D-LA and I-FABP in plasma harvested from the Shock+SGB+TM group were observably increased (*P*<0.05).

In **Figure 3C**, compared with sham group, the intestinal mucosa permeability to FD4 in the Shock group was significantly increased (*P*<0.05). By contrast, SGB and 4-PBA treatment abolished the hemorrhagic shock-induced hyper-permeability of intestinal mucosa (*P*<0.05). Moreover, TM reversed the favorable effect of SGB on intestinal mucosal permeability after acute hemorrhage.

### Effects of SGB and ERS related tool drugs on the expressions of initiator molecules of ERS

**Figure 4:**
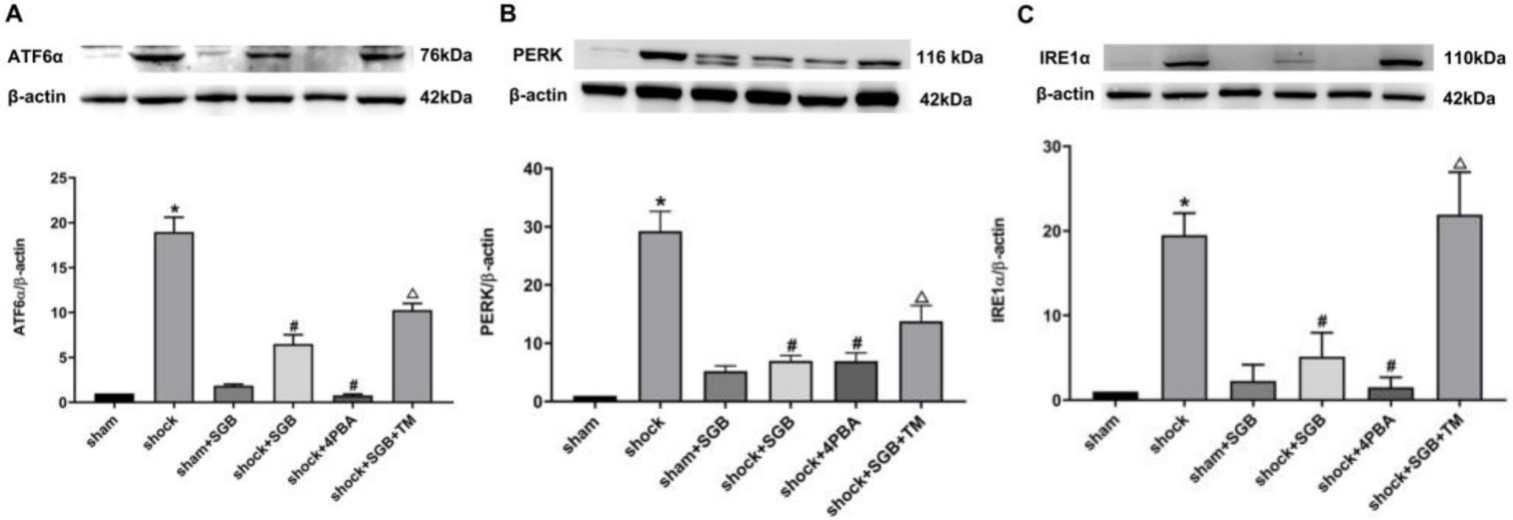
** SGB reduced expressions of ATF6α, PERK and IRE1α in intestinal tissue in rats after hemorrhagic shock.** Data are presented as mean ± SEM, with n = 3 for each group. The analysis of ANOVA showed that there was statistical difference among these six groups, followed by LSD test as follows: *P < 0.05 vs. the Sham group, ^#^*P* < 0.05 vs. the Shock group, ^Δ^*P* < 0.05 vs. the Shock+SGB group.

Results from **Figure 4** showed that hemorrhagic shock significantly unregulated the expressions of the ERS related initiator molecules ATF6α, PERK and IRE1α (*P*<0.05). Meanwhile, SGB and 4-PBA significantly decreased the expressions of ATF6α, PERK and IRE1α in the rats following acute hemorrhage (*P*<0.05). Furthermore, TM abolished the beneficial effects of SGB on these indices following hemorrhage (*P*<0.05)**.**

## Discussion

It has been shown that intestinal mucosal barrier dysfunction causes uncontrolled systemic inflammatory response and multiple organ dysfunctions during hemorrhagic shock, and ERS activation is involved in this process. Given that SGB improves the intestinal mucosal barrier dysfunction during hemorrhagic shock, this study employed SGB surgery, pharmacological method (ERS specific inhibitor or agonist) and molecular biology (protein expression) to explore the underlying mechanism by which SGB repairs the intestinal mucosal barrier damage via inhibition of ERS.

As one of the target organs of ischemia, intestinal tissue structure, intestinal villus height, submucosal thickness and muscular layer thickness, intestinal wet-dry ratio are indicators of intestinal injury in rats with hemorrhagic shock. In the present study, intestinal histological damage was evident, showing that the height of intestinal villi, the thickness of submucosa and muscular layer were decreased, and the intestinal wet-dry ratio was increased after hemorrhagic shock; SGB inhibited these adverse effects of hemorrhagic shock, reflecting the beneficial effect of SGB in reducing intestinal injury. Similarly, 4-PBA, a specific inhibitor of ERS, also plays a protective role in hemorrhagic shock-induced intestinal injury. In contrast, TM, a specific agonist of ERS, abolished the beneficial effect of SGB. These results indicate that the protective effect of SGB on intestinal injury after hemorrhagic shock is associated with ERS inhibition.

After hemorrhagic shock, the increase in intestinal permeability is a direct consequence of intestinal mucosal barrier dysfunction 8. It is not only an earliest pathophysiological change induced by hemorrhagic shock, but also a key link of intestinal infection. Therefore, our study primarily focused on the changes of intestinal permeability during hemorrhagic shock. It has been shown that intestinal clearance of FD4, an indicator of intestinal permeability 9, 10, was increased after hemorrhagic shock, which was inhibited by SGB, suggesting that SGB could improve intestinal mucosal permeability in rats with hemorrhagic shock. D-LA is a product that is released by a variety of gastrointestinal microorganisms through glycolysis in human. After intestinal barrier injury, intestinal mucosal permeability increases, and the D-LA produced by bacteria is consequently released into the blood stream. Therefore, the increased D-LA in the plasma also reflects the elevated intestinal mucosal permeability 11. I-FABP is released by mature intestinal cells which are located at the top of intestinal villi. Under physiological conditions, the content of I-FABP in peripheral circulation is very low, but it will increase rapidly after intestinal cell necrosis and inflammation 12. Thus, the levels of D-LA and I-FABP, alone or both, in circulation can reflect not only the degree of intestinal mucosal injury, but also the changes of intestinal mucosal permeability. In this study, we found that SGB resulted in significant decreases in the D-LA and I-FABP levels after hemorrhagic shock, indicating a protective effect of SGB on the intestinal mucosal barrier function. Furthermore, the results showed that 4-PBA played a beneficial role similar to that of SGB, while TM abolished the beneficial role of SGB, suggesting that SGB protects intestinal mucosal permeability through the inhibition of ERS.

Studies 13 have shown that ischemia and hypoxia cause energy metabolism disorder, calcium overload and free radical production in tissue cells after hemorrhagic shock. Misfolded and unfolded proteins gradually accumulate in the endoplasmic reticulum, resulting in endoplasmic reticulum dysfunction and ERS. At the same time, ERS also triggers unfolded protein response (UPR) to restore endoplasmic reticulum homeostasis through three endoplasmic reticulum transmembrane sensors such as ATF6α, IRE1α and PERK 14. When UPR is dysfunctional, the hyper secretory cells in the small intestine, such as Paneth cells and goblet cells, are particularly prone to abnormalities and high expression of UPR-related genes 15. Recent study 16 has shown that excessive ERS and the impairment of UPR signal transduction cause inflammatory bowel disease mainly through reduction of intestinal epithelial cell apoptosis and excessive inflammatory response.

To clarify the inhibitory effect of SGB on ERS in improving intestinal mucosal permeability in rats with hemorrhagic shock, we further observed the effects of SGB on the expression of ATF6α, IRE1α and PERK proteins, three indicators for ERS signaling pathways in intestinal tissue of rats with hemorrhagic shock. We found that hemorrhagic shock increased the expressions of ATF6α, IRE1α and PERK in intestinal tissues. However, SGB inhibited the upregulated protein expression, suggesting that SGB reduced excessive intestinal ERS caused by hemorrhagic shock. Unsurprisingly, ERS blocker 4-PBA inhibited excessive intestinal ERS induced by hemorrhagic shock, and ERS agonist TM abolished the inhibitory effect of SGB on the expressions of ATF6α, IRE1α and PERK in intestinal tissues. These results again demonstrated that excessive ERS is a major cause of intestinal mucosal barrier injury during hemorrhagic shock, and that SGB protects the intestinal mucosal barrier by inhibiting ERS.

In this study, we used the model of conscious hemorrhagic shock. The advantage of this model is that the operation was performed under inhalation anesthesia, and the animals were naturally awakened. Because the animals lose blood in the conscious state, this model is more likely to mimic the acute blood loss in human. Moreover, the current finding that SGB improved the intestinal mucosal barrier in conscious rats with hemorrhagic shock is consistent with the results of acute blood loss in anesthetized rats 5 which advances the experimental method in studying the hemorrhagic shock in conscious animals.

Interestingly, in the present study, SGB, 4-PBA or TM treatments did not affect the MAP in rats following hemorrhagic shock. The results suggest that SGB treatment does not affect the benign stress on the blood pressure during acute hemorrhage, which may be beneficial for ensuring the blood perfusion of vital organs and for further expanding its clinical application. These results also indicate that the favorable effect of SGB may be unrelated to MAP recovery, and the related mechanism of SGB action needs to be investigated in the future.

In summary, either SGB or ERS inhibitor 4-PBA reduced intestinal injury, improved intestinal permeability and decreased the expressions of ATF6α, IRE1α and PERK in hemorrhagic shock rats. The beneficial role of SGB was abolished by ERS specific agonist TM. These findings indicate that SGB attenuates intestinal mucosal barrier injury induced by hemorrhagic shock through inhibiting ERS. This study provides new evidence for expanding the clinical application of SGB, and may represent new therapeutic strategy for the prevention and treatment of intestinal mucosal injury after shock.

## Conclusion

The protective effect of SGB on intestinal mucosal barrier is related to the inhibition of intestinal mucosal excessive ERS caused by hemorrhagic shock.

## References

[1] Cannon JW (2018). Hemorrhagic shock. N Engl J Med.

[2] Leung CH, Caldarone CA, Wang F, Venkateswaran S, Ailenberg M, Vadasz B, Wen XY, Rotstein OD (2015). Remote ischemic conditioning prevents lung and liver injury after hemorrhagic shock/Resuscitation: potential role of a humoral plasma factor. Ann Surg.

[3] Wells JM, Brummer RJ, Derrien M, MacDonald TT, Troost F, Cani PD, Theodorou V, Dekker J, Meheust A, de Vos WM, Mercenier A, Nauta A, Garcia-Rodenas CL (2017). Homeostasis of the gut barrier and potential biomarkers. Am J Physiol Gastrointest Liver Physiol.

[4] Haq S, Grondin J, Banskota S, Khan WI (2019). Autophagy: roles in intestinal mucosal homeostasis and inflammation. J Biomed Sci.

[5] Zhang J, Lin XR, Zhang YP, Zhang LM, Du HB, Jiang LN, Zhao ZG, Niu CY (2019). Blockade of stellate ganglion remediates hemorrhagic shock-induced intestinal barrier dysfunction. J Surg Res.

[6] Chen Y, Guo L, Lang H, Hu X, Jing S, Luo M, Xu G, Zhou Z (2018). Effect of a stellate ganglion block on acute lung injury in septic rats. Inflammation.

[7] Rao G, Yadav VR, Awasthi S, Roberts PR, Awasthi V (2016). Effect of liposome-encapsulated hemoglobin resuscitation on proteostasis in small intestinal epithelium after hemorrhagic shock. Am J Physiol. Gastrointest Liver Physiol.

[8] Salim SY, Soderholm JD (2011). Importance of disrupted intestinal barrier in inflammatory bowel diseases. Inflamm Bowel Dis.

[9] Wattanasirichaigoon S, Menconi MJ, Delude RL, Fink MP (1999). Lisofylline ameliorates intestinal mucosal barrier dysfunction caused by ischemia and ischemia/reperfusion. Shock.

[10] Xia XM, Wang FY, Xu WA, Wang ZK, Liu J, Lu YK, Jin XX, Lu H, Shen YZ (2010). CXCR4 antagonist AMD3100 attenuates colonic damage in mice with experimental colitis. World J Gastroenterol.

[11] Liu W, Wang XH, Yang XJ, Zhang XY, Qi WJ (2016). Intestinal barrier dysfunction and its related factors in patients with sepsis. Zhonghua Yi Xue Za Zhi.

[12] Piton G, Capellier G (2016). Biomarkers of gut barrier failure in the ICU. Curr Opin Crit Care.

[13] Jian B, Hsieh CH, Chen J, Choudhry M, Bland K, Chaudry I, Raju R (2008). Activation of endoplasmic reticulum stress response following trauma-hemorrhage. Biochim Biophys Acta.

[14] Hosoi T, Ozawa K (2009). Endoplasmic reticulum stress in disease: mechanisms and therapeutic opportunities. Clin Sci (Lond).

[15] McGuckin MA, Eri RD, Das I, Lourie R, Florin TH (2010). ER stress and the unfolded protein response in intestinal inflammation. Am J Physiol Gastrointest Liver Physiol.

[16] Kaser A, Lee AH, Franke A, Glickman JN, Zeissig S, Tilg H, Nieuwenhuis EE, Higgins DE, Schreiber S, Glimcher LH, Blumberg RS (2008). XBP1 links ER stress to intestinal inflammation and confers genetic risk for human inflammatory bowel disease. Cell.

